# The Impact of Tiered-Pricing Framework on Generic Entry in Canada

**DOI:** 10.34172/ijhpm.2020.215

**Published:** 2020-11-16

**Authors:** Wei Zhang, Huiying Sun, Daphne P. Guh, Larry D. Lynd, Aidan Hollis, Paul Grootendorst, Aslam H. Anis

**Affiliations:** ^1^Centre for Health Evaluation and Outcome Sciences, Vancouver, BC, Canada.; ^2^School of Population and Public Health, University of British Columbia, Vancouver, BC, Canada.; ^3^Faculty of Pharmaceutical Sciences, University of British Columbia, Vancouver, BC, Canada. 4; ^4^Department of Economics, University of Calgary, Calgary, AB, Canada.; ^5^Leslie Dan Faculty of Pharmacy, University of Toronto, Toronto, ON, Canada.

**Keywords:** Tiered-Pricing Framework, Price-Cap Regulation, Generic Drug, Generic Entry, Canada

## Abstract

**Background:** Generic drug prices have been capped at specified percentages of the interchangeable branded drug’s price by the Canadian provincial public drug plans since 1993. The Pan-Canadian Pharmaceutical Alliance, formed as a coalition by the provinces/territories in Canada, implemented an alternative approach, a tiered-pricing framework (TPF) for new generic drugs on April 1, 2014, under which the percentage varies with the number of generic firms in each market. We evaluate the impact of the TPF on generic entry, ie, listing in public drug plans in Canada.

**Methods:** Our study compared the pre-TPF period (01/01/2012-03/31/2014) with the TPF period (04/01/2014- 06/30/2016). Prescription drugs from nine provincial public drug plans were grouped into a "market" if they had the same active ingredient and strength, route of administration, and dosage form. Each "market" was contestable by generics and met the eligibility criteria for TPF. At the "market" level, Cox proportional-hazards models with time-varying covariates were used to measure the impact of the TPF on the first generic listing in any provincial public drug plan in Canada relative to the first launch date worldwide.

**Results: **A total of 189 markets in Canada were selected for the analyses. Generic drugs in small markets were more likely to be listed in Canada during the TPF period compared to the pre-TPF period (hazard ratio [HR], 95% CI: 3.81, 1.51-9.62). There was no significant difference in generic drug listings in large markets between the two policy periods.

**Conclusion:** TPF speeds up generic entry in small markets and generates the benefits of generic competition while avoiding the pitfalls of the previously employed price-cap regulations.

## Background

**Key Messages**

**Implications for policy makers**

- The Pan-Canadian Pharmaceutical Alliance implemented a tiered-pricing framework (TPF) for new generic drugs in 2014 whereby the reimbursement price declines with the number of generic firms supplying the market; previously, the reimbursement price did not vary with the number of competitors.
- Our study shows that the TPF speeds up the entry of generic drugs (listing in provincial public drug plans in Canada) in small markets and generates the benefits of generic competition while avoiding the drawbacks of previous price-cap regulations.
- The TPF may be adapted in other settings seeking to control the prices for off-patent drugs as it encourages generic entry and competition.

**Implications for the public**

Generic competition is important for lowering drug costs for governments and patients and for creating greater access to drugs for patients. Generic drug prices have been capped at specified percentages of the interchangeable branded drug’s price in Canada since 1993. The Pan-Canadian Pharmaceutical Alliance implemented a tiered-pricing framework (TPF), an alternative approach, for new generic drugs on April 1, 2014, under which the percentage varies with the number of generic firms supplying the market. Our study shows that compared with previous price-cap regulations, the TPF speeds up the entry of generic drugs (listing in provincial public drug plans in Canada) in small markets and generates the benefits of generic competition. The TPF may be adapted in other settings or countries seeking to control the prices for off-patent drugs as it encourages generic entry and competition.

In 2017, the generic drug market accounted for 77.2% of prescription drug claims and 31.3% of public drug program spending in Canada.^1^ Canada has enacted several policies over time to promote its generic drug sector, which is important in lowering costs for drug plans and patients, and also improving drug access for patients. As early as 1923, the Patent Act included provisions to allow for generic manufacturing.^2-4^ While the 1923 Act did not result in a generic manufacturing industry in Canada, the compulsory licensing provisions of the Patent Act (1969) allowed for the importation of active ingredients. This increased the size of the generic market and the number of generic firms supplying it. The generic manufacturing industry in Canada had an accelerated growth rate until Bill C22 (1987) and subsequently Bill C91 (1992) ended compulsory licensing. It was an inevitable consequence of the North American Free Trade Agreement,^2-4^ which aimed to protect intellectual property rights of innovative pharmaceuticals. Since then, new trade agreements, including the United States-Mexico-Canada Agreement, have further strengthened intellectual property protections for innovative pharmaceuticals.^5^ Nonetheless, with provincial public drug plans in Canada seeking to save whenever they can substitute a branded product for a generic product, the generic products’ share of total number of accepted claims kept increasing from 67.4% in 2011 to 77.2% in 2017 and their share of drug program spending was slightly decreasing from 34.8% in 2011 to 31.3% in 2017.^1,6^

 Typically, Canadian federal policies aim to protect the intellectual property rights of companies marketing brand-name drugs whereas provincial policies work in the opposite direction by promoting generic competition. Lowering the cost of the provincially-funded prescription drug insurance plans has been the main motivation behind the provincial efforts which have had limited success: Canada has traditionally had some of the highest generic prices in the world.^7^ Furthermore, arbitrarily setting generic price-levels has led to generic firm exit as well as more concentrated markets in Canada.^8,9^ In recognition of the unanticipated and unintended consequences of provincially-led generic-pricing policies^7-9^ as well as the potential benefits of a national approach towards drug pricing, the pan-Canadian Pricing Alliance (pCPA) has evolved as a coordinating body of the provincial/territorial/federal governments that negotiates and sets prices for both the branded and generic sectors.^10^

**Figure 1:**
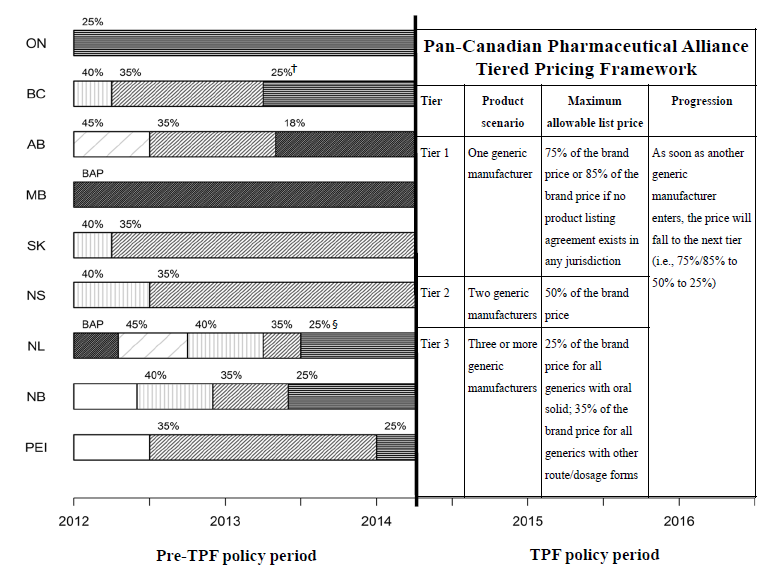
Pricing Policies for New Generic Drugs by Provinces From 2012 to 2016.^11,12^Abbreviations: ON, Ontario; BC, British Columbia; AB, Alberta; MB, Manitoba; SK, Saskatchewan; NS, Nova Scotia; NL, Newfoundland and Labrador; NB, New Brunswick; PEI, Prince Edward Island; BAP, best available price; TPF, tiered-pricing framework.

^†^35% non-oral solid generics; ^§^35% non-solid interchangeable generics; blank no MALP.

 Historically, the provincial public programs have used price caps, referred to as the “maximum allowable list price” (MALP),^11,12^ under which generic drug prices are capped at a fixed percentage of the respective branded product’s price. These MALP percentages were initially set at levels that were relatively high when compared to international standards.^13,14^ For instance, from 1993 to 1998 the public drug plan of the province of Ontario reimbursed generic drugs as high as 75% of the price of the interchangeable branded drug.^15,16^ After 2006, following Ontario’s lead, Canadian provinces reduced MALPs by varying amounts. By 2013, provincial MALP percentages were as low as 18% of the branded drug price in Alberta and as high as 35% in Saskatchewan (Figure 1),^11^ which is lower than the MALPs in countries such as Italy (80% MALP) and France (40% MALP).^13,14^ With the advent of the pCPA, a nationwide MALP of 18% was implemented in 2013 for several commonly prescribed generic drugs.^11,12^

 The MALP system has been criticized on several fronts. First, while the MALP is intended to set a maximum list price, many firms set their prices at the maximum allowable price and tend not to drop their prices over time.^15-17^ Second, drug plans do not have the information needed to set appropriate MALPs.^16,18,19^ If a MALP is too high, the drug plans overpay. If the MALP is too low, firms may fail to enter the market, thus delaying generic competition,^8^ or the existing firms may cease production.^9^ Previous studies have shown that lowering the MALP from 50% to 25% leads to a significantly lower probability of market entry (ie, listing in the public drug plan) and higher market exit by generic firms in Ontario, Canada.^8,9^ Other studies comparing multiple countries found that higher levels of price regulation delayed the time to generic entry.^20,21^ It is important to recognize that the effect of any MALP will depend on the specific institutional environment in which it is used. In Canada, generic firms have typically not priced below the ceiling. In other countries, complementary mechanisms may reduce generic prices; for example, in Italy while the MALP is at 80%, this is only the starting point for a negotiation between generic manufacturers and the Italian Medicines Agency, resulting in much lower prices.^22^

 To mitigate these problems, the pCPA adopted a tiered-pricing framework (TPF) for new generic drugs in Canada effective on April 1, 2014 to replace the MALP.^11,12^ Under the TPF, the maximum allowable reimbursement price starts off high and falls with the number of generic firms supplying the market. The underlying premise is that generic firms are expected to keep entering the market, driving the price down, as long as such entry is profitable. Entry ceases once the reimbursement price is at a level below which further entry is not profitable; this point represents the lowest feasible price that drug plans aspired to set under the old MALP system. This resembles systems used in numerous European countries, including Portugal and Austria.^13,14^

 Specifically, under the TPF, as soon as a generic firm enters a market in any jurisdiction in Canada, the price of the generic drug must fall to the next tier (ie, the MALP from 75%/85% to 50% to 25%) (Figure 1). For a small drug market expected to have only one or two generic supplying firms, the corresponding MALP percentages would be at 75% or 50%, which are higher than the percentages set by provincial public drug plans before the TPF (18% to 35% depending on provinces). On the other hand, for a large drug market expected to have three or more generic supplying firms, the MALP percentages would eventually reach 25%, which is similar to percentages before the TPF. Our hypothesis, therefore, was that the TPF would significantly speed up the generic entry (listing in the public drug plans) for small markets but have no or minimal impact on large markets. This TPF framework applies only to generic drugs launched on or after April 1, 2014. Generic drugs on the market prior to this date continue to be reimbursed at MALP values – using either the pCPA percentages for selected common molecules^12^ or the percentages set by each individual provincial drug plan.^11^

 While MALPs in Canada were similar in principle to those implemented in some European countries including France, Italy, and Switzerland, their impact with respect to price levels have been different.^7^ Moreover, the recent price hikes and efforts in the US to increase access to generic drugs and lower prescription drug costs^23,24^ show the importance of re-examining the MALP and evaluating its replacement with the TPF. Our study aimed to assess whether the TPF encourages generic entry into public markets in Canada.

## Methods

###  Data 

 We used several data sources. The National Prescription Drug Utilization Information System Database (NPDUIS), which is held at the Canadian Institute for Health Information, includes formulary data and claims data for all provinces in Canada except Quebec. The formulary data contain information on all drugs covered by the publicly-funded drug benefit programs in each province and track their formulary coverage start and end dates.^25^ The data is tabulated according to the Drug Identification Number (DIN), which uniquely identifies each drug by manufacturer, product name, active ingredient(s), dosage form (eg, extended release tablets, controlled release tablets, powder, liquids), route of administration (eg, oral, topical, intramuscular, rectal), and strength of active ingredient(s). Claims data are also available at the DIN level, aggregated from the claim level, and include the drug quantity and the cost claimed and accepted by the drug programs. Both formulary and claims data became available for nine out of ten provinces from April 1, 2010. Data from the province of Quebec and the three territories in Canada were not available and thus not included in our study.

 The Health Canada Drug Product Database^26^ was used to supplement NPDUIS data. We extracted data on drug schedule (eg, prescription drugs, over the counter drugs, narcotic), route of administration, dosage form, and active ingredient group number (10-digit number that identifies drug products with the same active ingredient(s) and ingredient strength(s)). The international generic drug launch date information of seven major countries including Canada, US, UK, France, Germany, Italy, and Japan was obtained from IQVIA (formerly IMS Health).

###  Study Design

 Our study period was January 1, 2012 to June 30, 2016. We compared generic entry in the period before the pCPA TPF, that is the pre-TPF MALP policy period (January 1, 2012 to March 31, 2014), to the TPF period (April 1, 2014 to June 30, 2016) (Figure 1). During the pre-TPF policy period, the MALPs varied over time and declined to 18%-35% at the end in all of the nine provinces except Ontario which had a constant 25% MALP. Thus, we evaluated the impact of the TPF in Canada as well as in Ontario to address the heterogeneity. To determine the eligibility of drugs for the pCPA TPF and to improve the comparison and consistency among the public drug plans in the different provinces, our selection criteria at the DIN level included: (1) DINs listed on at least one of the nine provincial formularies; (2) Prescription DINs; and (3) DINs on the drug list in the study of Patented Medicine Prices Review Board (PMPRB), “Alignment among Public Formularies in Canada” (full list available on their website).^27^ Exclusion criteria included (1) DINs under existing common molecules to which the pCPA applied a lower MALP^12^; and (2) DINs listed only under the new drug plans/programs launched during the study period.

 Following the method of determining applicable tier pricing applied by TPF,^12^ we grouped DINs if they had the same active ingredient(s) and strength, route of administration, and dosage form (tablets and capsules were considered the same). Such a drug group was defined as a “market.” Previous studies have also applied this method to define their observation units.^28,29^ We further selected “markets” that were eligible for generic entry if: (1) a market included branded DINs but did not include any associated generic DINs before January 1, 2012, the starting date of our study period; (2) a market’s first international generic launch (among the seven countries) was between April 1, 1993 and June 30, 2016 (if no generic product had been listed in public programs in Canada since its international launch before April 1, 1993, we deemed that it is very unlikely to occur later); and (3) a market’s branded DIN active period (the period between the first and last date of branded drug sales in public programs) was longer than one year and overlapped with the study period.

###  Variable Definitions

 Our unit of observation was at the “market” level. The main outcome was time to first generic entry (listing in public drug plans) in Canada, defined as the time from the first international generic launch to the first generic listing in any of the nine provincial formularies in Canada. Following the method of Costa-Font et al,^20^ we used the first international generic launch date (among the seven major countries) to indicate the eligibility timing for generic entry in Canada.

 We controlled for two potential confounding variables: market size and route/dosage formulation. Market size was defined as the annual branded drug sales in Canada (ie, total accepted claim cost of the branded DINs with the same active ingredients, route, and dosage form) before the first generic drug formulary listing in Canada, or the last available sales during the study time period if no generic drugs were listed. More specifically, for a given market, we used the value of its branded drug sales during a fixed one-year period, either one year right before the first generic drug formulary listing, or one year before its last observation date if no generic formulary listing occurred. Thus, the market size was fixed and did not change over time. We further classified markets into categories based on market size. The number of categories and the corresponding cut-off values were determined by exploratory analyses of generic entry in the two policy periods and different percentiles of market size (see detailed methods in Supplementary file 1). The larger market size was expected to have more generic supplying firms later. The markets with oral solid formulation were distinguished from those with other route/dosage formulations.

###  Analyses

 Cox proportional hazards models with time-varying covariates were used for the analysis.^30,31^ Model details including model specifications can be found in Supplementary file 2. The policy period was the time-varying variable in these models. We included the interaction between market size and policy period because as mentioned above, we expected a significant impact of the pCPA’s TPF on small markets but no or minimal impact on large markets. The “proportional” assumption was tested using weighted Schoenfeld residuals.^32^ The PROC PHREG procedure in the SAS software (version 9.4, SAS Institute Inc., Cary, NC) was used. As a secondary analysis, we assessed the impact of the TPF on the time to first generic entry in Ontario, which was the only province that had a constant MALP at 25% during the entire pre-TPF period (Figure 1). In addition, we have conducted sensitivity analyses using different cut-off values to categorize market size and different model specifications, and a sub-group analysis among the markets with oral-solid formulation.

## Results

**Figure 2:**
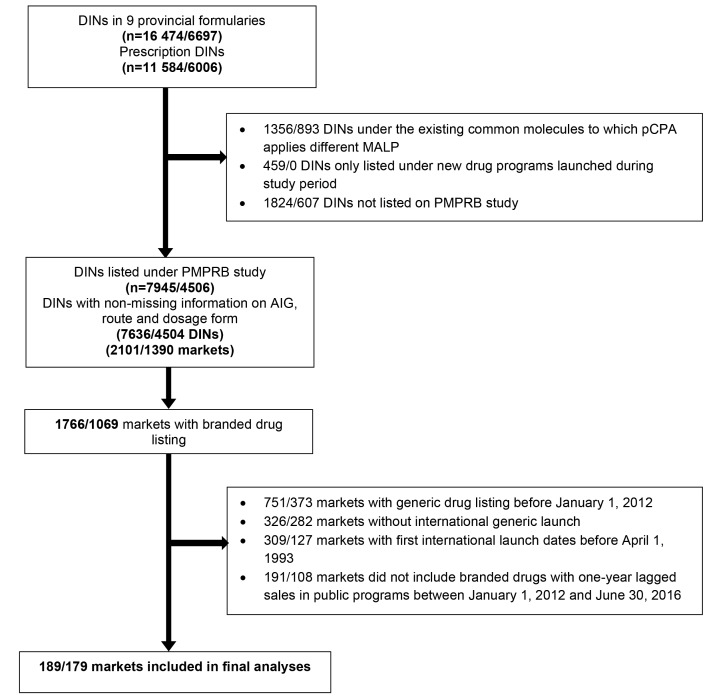
The Sample Selection Flowchart for Canada and Ontario, Respectively.The numbers shown as “the numbers in Canada/the numbers in Ontario.” Abbreviations: DIN, Drug Identification Number; pCPA, Pan-Canadian Pharmaceutical Alliance; MALP, maximum allowable list price; PMPRB, Patented Medicine Prices Review Board; AIG, Active Ingredient Group number (a 10 digit number that identifies drug products that have the same active ingredient(s) and ingredient strength (s)).

 A total of 189 markets (107 unique active ingredients) in Canada were selected for the analyses (Figure 2). Among them, 139 markets (73.5%) were classified as oral solid formulation. After exploratory analyses, we divided our study markets into large and small categories. The 40th and 50th percentile values of their market size were used and the corresponding market size values were $1.85 million and $3.04 million in Canada, respectively. Supplementary file 1 and Figure S1 show how cut-off values were determined. The route/dosage formulation and market size were highly correlated. For example, about 89.4% of the large markets (defined using the 40^th^ percentile value of the market size) in Canada were oral solid formulation.

**Figure 3:**
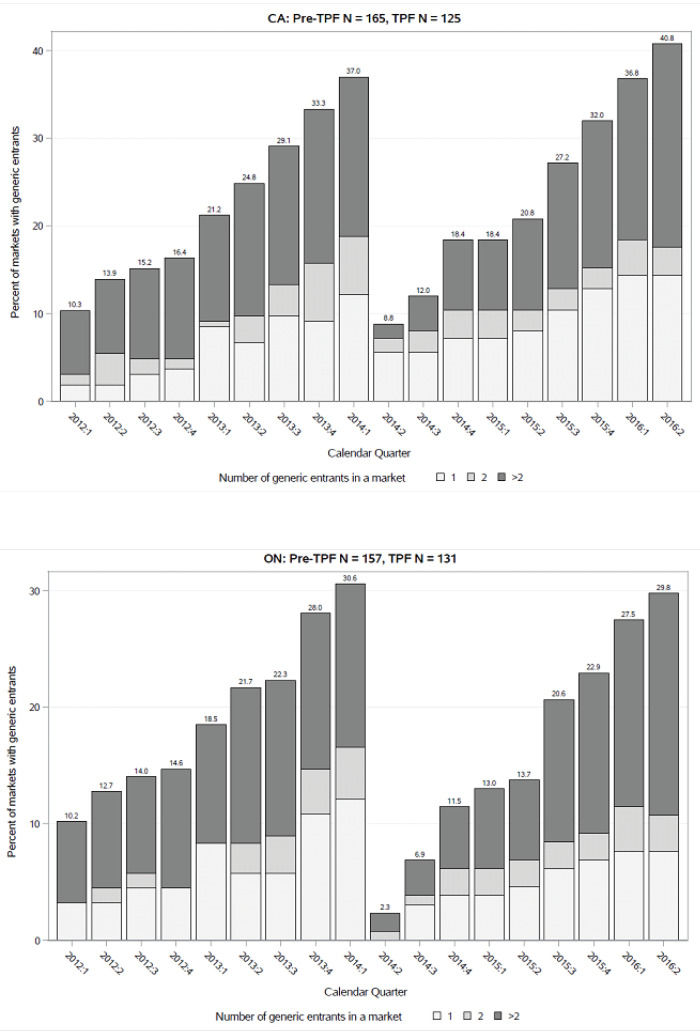
The Percentage of Markets With Generic Entrants, Cumulatively, Over Time (Calendar Quarters) During the Pre-TPF and TPF Periods. Abbreviations: TPF, tiered-pricing framework; CA, Canada; ON, Ontario.

 There were 112 markets with at least one generic listed in Canada during the entire study period. Among the markets with generic listings in the study period, the average time from the first international generic launch date to the first listing date in Canada was 4.80 years (standard deviation = 5.57 years). Figure 3 presents the percentage of markets with one, two, or more than two generic entrants, cumulatively, over time (calendar quarters) during the pre-TPF and TPF periods. There were 165 markets eligible for first generic entry during the pre-TPF period and 125 eligible markets during the TPF period in Canada. A total of 37.0% of the 165 markets had generic entrants (12.1% of markets with one generic entrant, 6.7% of markets with two generic entrants, and 18.2% with more than two entrants) at the end of pre-TPF period (the first quarter of 2014), compared with 40.8% at the end of the TPF period. The cumulative quarterly percentages show a higher and earlier first generic entry in Canada during the TPF period. The plots of the incidence rate of first generic entry (Figure S2 in Supplementary file 3) did not show any time-trends.

**Figure 4:**
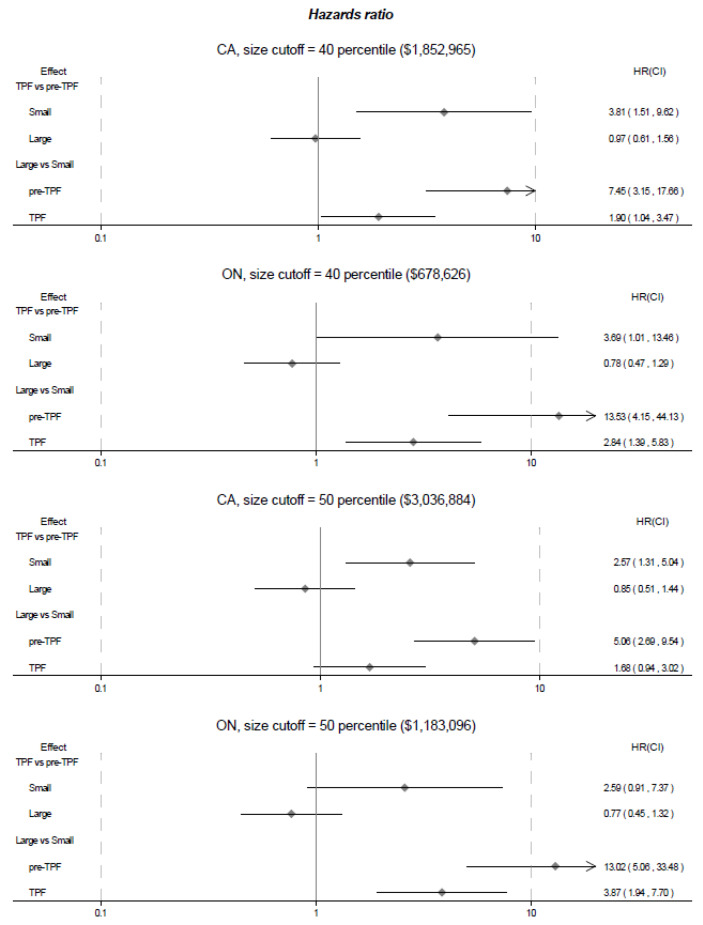
HRs From Cox Proportional-Hazards Models With Time-Varying Covariates. Abbreviations: TPF, tiered-pricing framework; CA, Canada; ON, Ontario; HR, hazard ratio; CI, confidence interval.

 Due to a high correlation between route/dosage formulation and market size, we did not adjust for route/dosage formulation in our main analysis but in a sensitive analysis later. Figure 4 presents hazard ratios (HRs) for the final models by the two different market size cut-off values at the 40th and 50th percentiles. The model results based on the 40th percentile of market size showed that generic drugs in small markets were more likely to be listed on formularies in Canada during the TPF period than during the pre-TPF period (HR of TPF vs. pre-TPF [95% confidence interval]: 3.81 [1.51, 9.62]). For large markets, there was no significant difference in generic entry between the pre-TPF and TPF periods (0.97 [0.61, 1.56]). Generic drugs in the large markets were more likely to be listed than those in the small markets in both the pre-TPF period (HR of large markets vs. small markets: 7.45 [3.15, 17.66]) and the TPF period (1.90 [1.04, 3.47]). The HR in the TPF period was lower than that in the pre-TPF period because of the relatively higher hazard of generic entry in the small markets in the TPF period.

 Secondary analysis assessing the impact of the TPF on generic entry in Ontario shows similar results. Generic drugs in small markets were more likely to be listed on the Ontario formulary during the TPF period than the pre-TPF period (ie, the “25% MALP for all”) (3.69 [1.01, 13.46]). The TPF did not affect generic entry in large markets (0.78 [0.47, 1.29]).

 When using the 50th percentile of market size to determine the large and small markets, the impact magnitude of TPF on generic entry for small markets in Canada was smaller. In Ontario, the impact for small markets was no longer statistically significant. Table S1 in Supplementary file 3 presents all model parameters. No substantial deviations from the assumption on proportional hazards was observed in any of our analyses (Table S2 in Supplementary file 3). As a sensitivity analysis, we repeated our analyses by adjusting for route/dosage formulation and conducted analyses among the markets with oral-solid formulation. The findings were consistent (Figures S3-S5 in Supplementary file 3).

 Table S3 in Supplementary file 3 presents the proportion of second and third entry among markets with first generic entry during the pre-TPF and TPF periods, the time to second generic entry from the first generic entry and the time to third generic entry from the second generic entry. Both second and third generic entries were faster in the TPF period than the pre-TPF period.

## Discussion

 Given that provincial drug plans do not have any informational advantages over other market participants, setting the level of the MALP was based on prior beliefs and guesswork. These likely included beliefs about generic drug production costs, comparison of prices across different international markets, and concerns regarding security of supply and potential shortages. Consequently, “the one MALP for all drugs” could be set either below the generic manufacturer’s reservation price, in which case there was no entry, or above this price, in which case there were excess profits. The TPF is an innovative generic pricing policy that can mitigate these problems by effectively using the generic manufacturers’ profit motive to determine the lowest feasible price.

 In a scenario where direct comparison of the MALP versus no-MALP is not feasible, our study examined the impact of the alternative, the TPF compared with the “one MALP for all drugs” policy. We found that the TPF sped up the generic entry in Canada for small markets, defined by an annual $1.85 million branded drug sales before the first generic entry in the public drug plans of nine provinces, while not affecting the generic entry in large markets. These results are consistent with our hypothesis.

 There are other different approaches used to determine generic pricing internationally. The use of tendering has frequently been proposed as a way to get the lowest prices.^33^ However, the lower price has been found to be associated with a higher risk of drug shortages.^29^ The chief advantage of TPF is that it provides incentives for generic companies to challenge patents, in the absence of which patent monopolies may be sustained.^34^ For example, in the first six months of 2020, 40 new cases related to generic entry were litigated in Canadian courts.^35^ Additionally, TPF allows for multiple suppliers, reducing the risk of shortages. Zhang et al have recently found that markets with a single generic manufacturer were more likely to be in shortage and newer generic drugs (a binary variable using the starting date of TPF, April 1, 2014, as the cut-off) were less likely to be in shortage.^36^ The evidence suggests that the TPF could reduce drug shortages especially for small markets with a single generic manufacturer in a long term.

 In addition, many countries apply reference pricing approach for off-patented drugs, that is, categorizing drugs including branded and generic drugs into a group and applying the same maximum reimbursement price (eg, the lowest price among them) for this group.^13,14,37,38^ The definition of the reference pricing group varies by countries. In addition to any other relevant criteria, drugs could be grouped by active ingredient, eg, in France and Italy, or by similar therapeutic effect, eg, in Germany, Netherland and the province of British Columbia in Canada.^13,14,37-39^ The intention of the reference pricing is to promote the price competition among products in the same group and thus the total expenditures. For example, it has shown the reference pricing was effective in reducing the price.^40,41^ However, Brekke et al reviewed the impact of reference pricing on generic entry and found that the evidence was mixed – impact ranged from positive, negative, to no effect.^42^ In Canada, British Columbia is the only one province that applies this approach for their public drug plan. Its reference drug program was introduced in 1995 for only three therapeutic classes of drugs (an additional two were added in 1997), and then did not change over the study period.^39,43^ Thus, our study results in entire Canada and in Ontario only were not affected by the existing reference drug program implemented in British Columbia.

 As already noted, when the MALP is set too low, generic firms are discouraged from entering the market, which could either deter or delay generic competition. Zhang et al found that lowering the MALP from 50% to 25% led to a lower probability of generic entry in Ontario.^8^ Two other studies comparing multiple countries found that higher levels of price regulation delayed the time to generic entry.^20,21^ Our study contributes to this literature and provides evidence that a less regulated pricing policy stimulates generic entry.

 Due to differing international patent expiry dates and patent challenges, it is empirically difficult to determine when a market is contestable by generics therefore measure the delay in entry attributable to pricing regulations. Often, multiple patents exist for a single branded drug and it is challenging to determine the relevant patent expiry date for a specific market. As such, we followed the method reported by Costa-Font et al^20^ by using the first generic launch date in seven major pharmaceutical markets (including Canada) to indicate the eligible timing for generic entry.

 One of our study limitations was that we only included the claims and formulary data from nine Canadian provinces – Quebec and the three territories were not considered due to data unavailability. However, there is a high degree of alignment in formulary coverage among the public drug plans of different provinces in Canada so that our study has included the majority of drugs.^44^ Furthermore, although Quebec did not participate in the pCPA policy until October 2015,^45,46^ the province has been requiring the generic manufacturers to provide the lowest price available in other provinces (the “best available price” pricing policy).^47^ Thus, we believe that the impact of the missing data is negligible.

 Another potential limitation is that our results may be a reflection of changes in the generic drug approval time by Health Canada over the study period. Health Canada requires an abbreviated new drug submission from generic drug manufactures for approval. The guidance on the management of drug submissions and applications, including the performance standards for drug submission/application review, has not significantly changed since its first major revision in 1993 until 2019.^48^ In addition, we requested the 2016-2017 Therapeutic Products Directorate’s Annual Drug Submission Performance Report from Health Canada,^49^ which included the actual abbreviated new drug submission approval times over the past five years. The report showed that the approval times were comparable during our two policy periods.

 In addition to investigating the impact of TPF on generic entry, it is important to examine the impact of TPF on the total expenditures on generics. Total expenditures on generics during the two different pricing policy periods (MALP and TPF) could not be directly compared because our study “markets” were different in the two periods. A recent PMPRB report showed generic pricing has kept dropping over time from 2010 to 2018 including the TPF period, which indirectly suggests a decrease in total expenditures on generics during the TPF period.^50^ Future studies are required to examine a longer term impact of TPF on generic entry and total expenditures.

 In an effort to control drug costs, Canada was among the first countries to adopt pro-generic drug policies. During the 1970s and 1980s, it was able to operate with impunity by exercising the compulsory licensing provisions of the Patent Act. This was in sharp contrast to developments in the US, where the US Drug Price Competition and Patent Term Restoration Act provided longer patent terms for branded firms.^51^ However, the implementation of the Canada-US Free Trade Agreement in 1988 resulted in the end of widespread compulsory licensing of pharmaceutical patents in Canada and since then the rate of new generic drug launches is line with the rest of the developed world economies. This policy reversal at the federal level did not however inhibit Canadian provinces from continuing in their efforts to obtain lower generic drug prices. They implemented MALP policies which did not prove to be effective and created unintended consequences.^7-9^ TPF is the latest incarnation of these policies to achieve the benefits of generic competition while avoiding the pitfalls of previous MALP policies.

 Our study has important policy implications for other countries. In the US, due to few or no competitors, the prices for some off-patent drugs have increased by more than 1000% in recent years, for example, a 3,100% price increase of Nitroprusside for hypertension in 2012 to 2015 and a 1400% price increase of Lomustine for brain tumors and Hodgkin’s lymphoma in 2013 to 2018.^23^ Policy makers are seeking approaches to reduce the massive price increases for the off-patent drugs with insufficient competition.^23,24^ To control drug costs, some European countries are still applying one MALP for all generic drugs.^13,14^ A TPF by varying MALP levels based on the number of competitors might serve as an option for these countries to help encourage generic entry and competition.

## Conclusion

 Our findings suggest that TPF speeds up the generic entry for small markets without affecting large markets and generates the benefits of generic competition while avoiding the pitfalls of the previously employed price-cap regulations. The TPF is demonstrated to be an effective approach regulating generic drug prices, given that Canada appears unwilling to consider eliminating price regulation on generic drugs altogether.

## Acknowledgments

 WZ acknowledges the support by the Michael Smith Foundation for Health Research Scholar Award.

## Ethical issues

 Ethics approval was not required as this study focused on drug data.

## Competing interests

 AH has received compensation for having provided expert reports relating to patent litigation on behalf of Apotex, Mylan and Pharmascience. PG has received compensation for: expert reports relating to patent litigation on behalf of Apotex and Teva, and reports commissioned by the Canadian Generic Pharmaceutical Association. None of these reports are related to the topic of the paper. All other authors declare no conflict of interest.

## Authors’ contributions

 WZ, AHA, AH, PG, and LDL are investigators of the operating grant funded by the Canadian Institutes of Health Research. WZ initially conceived and designed the research and all authors made substantial contributions to the further development of the research question and the design of the work; WZ requested for the data and HS and DPG linked all data from different sources together and analyzed the data; All authors contributed to the result interpretation; WZ drafted the paper and other authors revised it critically for important intellectual content; All authors approved the final version of the paper to be published and agreed to be accountable for all aspects of the work in ensuring that questions related to the accuracy or integrity of any part of the work are appropriately investigated and resolved.

## Authors’ affiliations

^1 ^Centre for Health Evaluation and Outcome Sciences, Vancouver, BC, Canada. ^2^School of Population and Public Health, University of British Columbia, Vancouver, BC, Canada. ^3^Faculty of Pharmaceutical Sciences, University of British Columbia, Vancouver, BC, Canada. ^4^Department of Economics, University of Calgary, Calgary, AB, Canada. ^5^Leslie Dan Faculty of Pharmacy, University of Toronto, Toronto, ON, Canada.

## Funding

 This work was supported by the Canadian Institutes of Health Research Project Grant (PJT-153280). The funder had no role in design and conduct of the study, data collection, data management, data analysis and interpretation, preparation, review and approval of the manuscript.

## Supplementary files


Supplementary file 1 contains Figure S1. Determining Number of Categories and the Cut-off Values for Market Size.

Supplementary file 2. Cox Proportional Hazards Model With Time-Varying Covariates.

Supplementary file 3 contains Figures S2-S5 and Tables S1-S3.
