# Supplementary material for: The Impact of Tiered-Pricing Framework on Generic Entry in Canada
Source: Int J Health Policy Manag. 2020 Nov 16;11(6):768–76. doi: 10.34172/ijhpm.2020.215 (PMC9309918; doi:10.34172/ijhpm.2020.215)
Supplement: Supplementary file 1 — contains Figure S1. Determining Number of Categories and the Cut-off Values for Market Size. [file ijhpm-11-768-s001.pdf]

Supplementary file 1. Determining Number of Categories and the Cut-off Values for Market Size

We have conducted exploratory analyses by running the models by dividing all markets into five equal categories (quintiles), four categories (quartiles), three categories (tertiles) and two categories (median or 50 percentile). The results (Figure S1 below) show that we do not have power for three or more categories due to the small sample size and the results based on quintiles (5 categories), quartiles (4 categories) and 50 percentile (2 categories) support having two number of categories (small vs. large). The generic entry in small markets is significantly more likely during the tiered pricing framework (TPF) period than during the pre-TPF period. Based on the results from 5 categories, we also used 40 percentile as the cut-off for two categories.

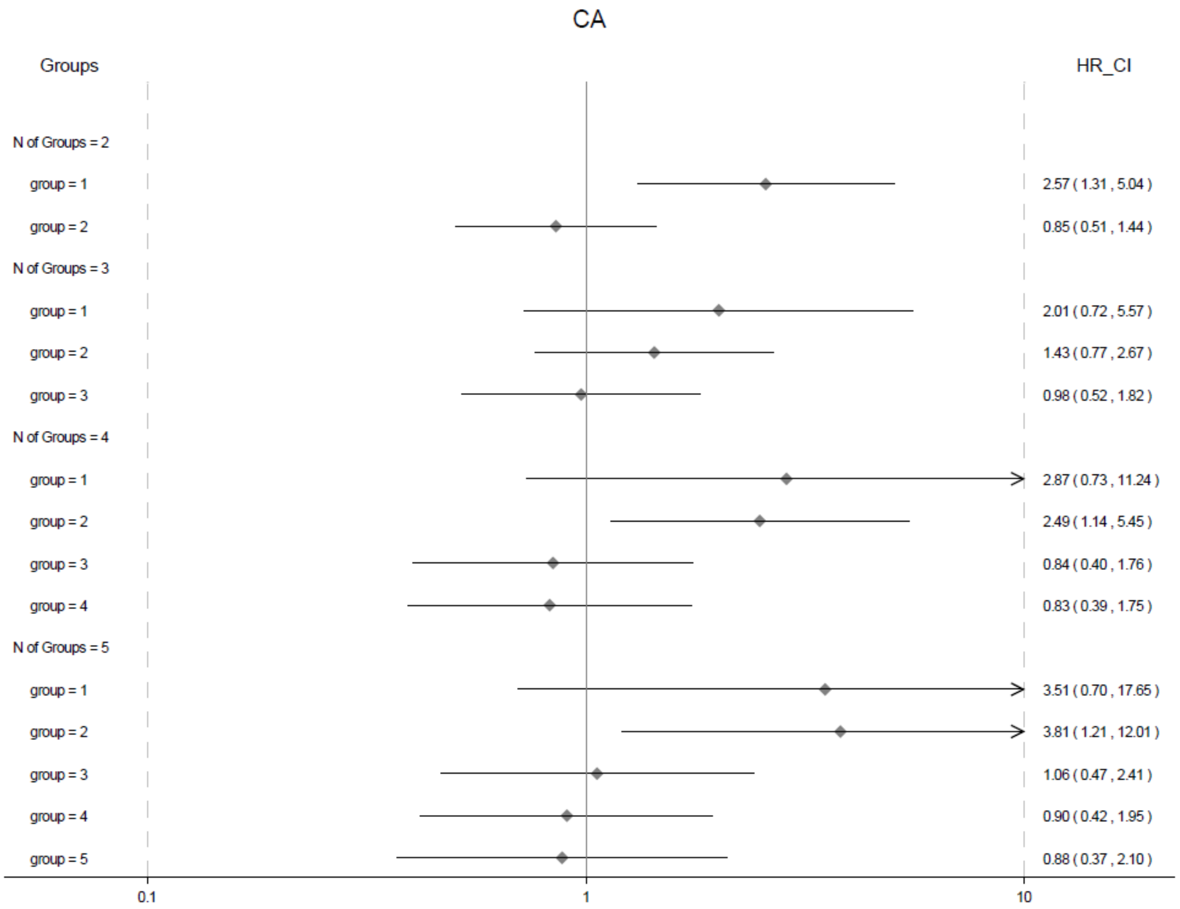

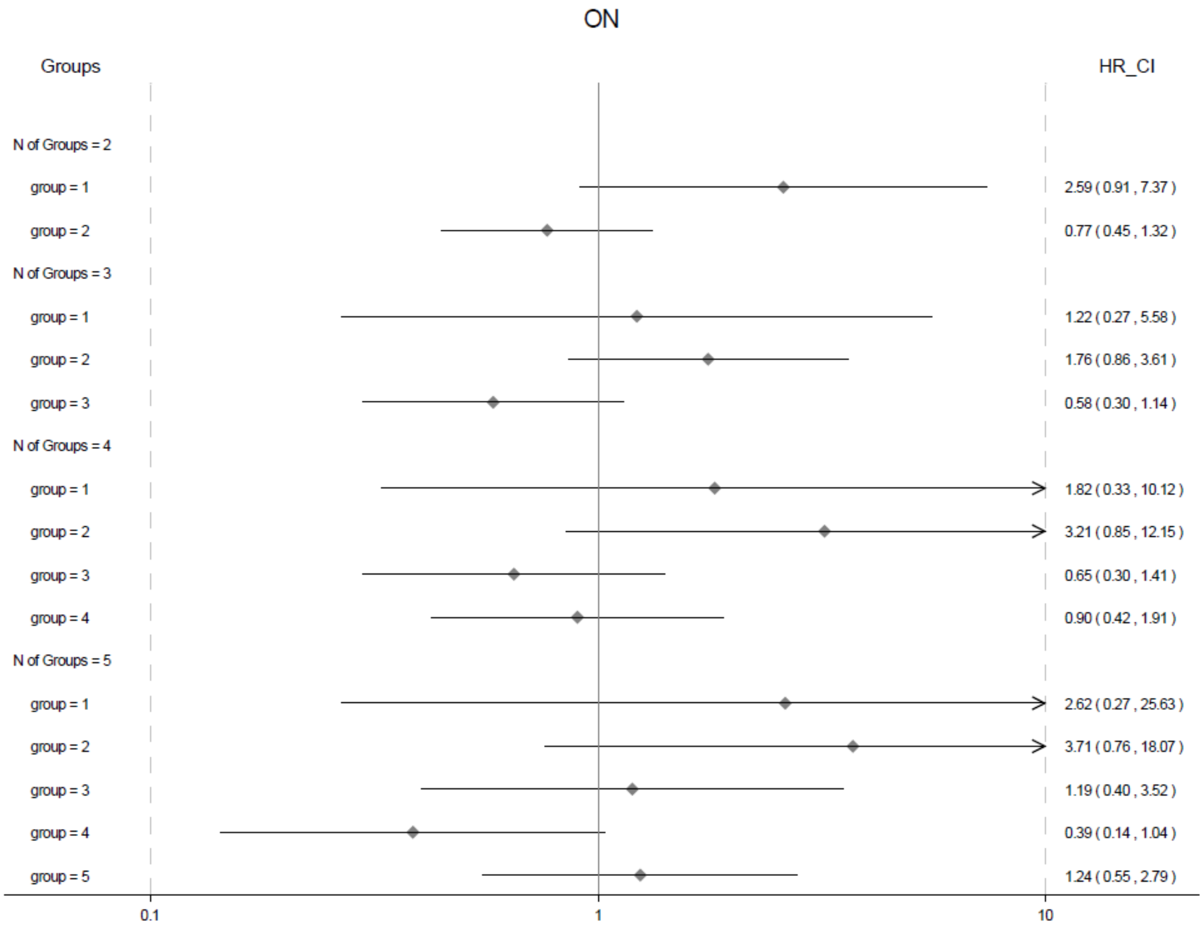

Figure S1. Exploratory Analyses of Determining Number of Categories and the Corresponding Cut-Off Values for Market Size. Abbreviation: CA, Canada; ON, Ontario; HR, hazard ratio; CI, confidence interval.
