# Supplementary material for: The Impact of Tiered-Pricing Framework on Generic Entry in Canada
Source: Int J Health Policy Manag. 2020 Nov 16;11(6):768–76. doi: 10.34172/ijhpm.2020.215 (PMC9309918; doi:10.34172/ijhpm.2020.215)
Supplement: Supplementary file 2 — Cox Proportional Hazards Model With Time-Varying Covariates. [file ijhpm-11-768-s002.pdf]

Supplementary file 2. Cox Proportional Hazards Model With Time-Varying Covariates

Cox proportional hazards modes are commonly used regression models in survival analysis. In survival analysis, the response variable is the survival time (or time to an event), which is defined as the duration from the beginning of an observation period to the time of an event occurrence or the end of the observation, denoted by T.[1,2] The survival function of T is defined as the probability of observing a survival time greater than some value, t, denoted by $S(t) = P(T > t) = 1 - F(t)$, where $F(t)$ is the cumulative density function of the survival time. The hazard function, the risk of failure (or event occurrence) in a moment after time t, is defined as $h(t) = \frac{f(t)}{S(t)}$, where $f(t)$ is the density function of the survival time. Suppose there are n subjects. Let $x_{lk}(t), k = 1,2, \dots \dots p, l = 1,2, \dots \dots, n$, denote the value of the kth covariate for subject l at time t and

$$\boldsymbol{x}'_{\boldsymbol{l}}(\boldsymbol{t}) = [\boldsymbol{x}_{\boldsymbol{l1}}(\boldsymbol{t}), \boldsymbol{x}_{\boldsymbol{l2}}(\boldsymbol{t}), \dots \dots, \boldsymbol{x}_{\boldsymbol{lp}}(\boldsymbol{t})]$$

denote the vector for the p covariates. Cox proportional hazard models with time-varying covariates assume that the hazard function for given time-varying covariates is the product of two functions: $h(t|x(t), \beta) = h_0(t)e^{x'(t)\beta}$, where β is a vector of unknown regression parameters, and $h_0(t)$ is a function of survival time.[1,2] The estimators of the coefficients are obtained by maximizing the partial likelihood function

$$\boldsymbol{l_p}(\boldsymbol{\beta}) = \prod_{\boldsymbol{l=1}}^{\boldsymbol{n}} \left[\frac{\boldsymbol{e}^{\boldsymbol{x}'_{\boldsymbol{l}}(\boldsymbol{t}_{(\boldsymbol{l})})\boldsymbol{\beta}}}{\sum_{\boldsymbol{j} \in \boldsymbol{R}(\boldsymbol{t}_{(\boldsymbol{l})})} \boldsymbol{e}^{\boldsymbol{x}'_{\boldsymbol{j}}(\boldsymbol{t}_{(\boldsymbol{l})})\boldsymbol{\beta}}} \right]^{\boldsymbol{c_l}}$$

where $c_l, l = 1,2, \dots \dots, n,$ is a censor indicator, $t_{(l)}$ is ordered survival time of subject l, and $R(t_{(l)})$ is the set of all subjects in the risk at time $t_{(l)}$.

In current study, our outcome is the time duration from the first international generic launch among the seven major countries (the beginning of the observation) to the first generic listing in Canada (event). We use the Cox proportional hazards model with time-varying covariates to estimate in

the ratio of hazards of event occurring (the first generic formulary listing in Canada) in the two policy periods (post vs pre). For a given market (subject), the duration might be only in pre-TPF (if both the international generic launch and formulary listing in Canada were before TPF), only in post-TPF (if the international generic launch was after TPF), or cross the both pre- and post-TPF (if the international generic launch was before TPF and the first formulary listing in Canada was after TPF or the generic drugs had not be listed in Canada during the study period, i.e., the market experienced two policy periods before a generic drug formulary was listed in Canada). Moreover, the time from the international generic launch to TPF was in a random fashion as the date of international generic launch was random. Thus, in our model, the policy period is a time-varying covariate, denoted as $x_1(t)$ (pre = 0, post = 1). We adjust for market size (not time-varying), denoted as x_2 (small = 0, large = 1), and include the interaction between market size and policy period, denoted as $x_3(t)$. Under the proportional hazard assumption, the hazard function can be written as

$$\boldsymbol{h(t|x(t),\beta) = h_0(t)e^{\beta_1 x_1(t)+\beta_2 x_2+\beta_3 x_3(t)}}.$$

The estimated hazard ratio for policy period (post vs pre) of large markets is

$$\boldsymbol{HR(t, x_1(t) = 1\ vs\ x_1(t) = 0|x_2 = 1) = \frac{h_0(t)e^{\beta_1+\beta_2+\beta_3}}{h_0(t)e^{\beta_2}} = e^{\beta_1+\beta_3}},$$

while the estimated hazard ratio for policy period (post vs pre) of small markets is

$$\boldsymbol{HR(t, x_1(t) = 1\ vs\ x_1(t) = 0|x_2 = 0) = \frac{h_0(t)e^{\beta_1}}{h_0(t)} = e^{\beta_1}}.$$

Since the policy period was a calendar time dummy variable, it might also capture some calendar time-related effects other than the policy. In our analyses, we assumed that the dependent variable (duration from the international generic launch to the first formulary listing in Canada) and the calendar time of generic formulary listing were independent. We calculated the incident rate (quarterly) of the first generic formulary listing based on the observed data. The plots of the

incidence rate of first generic entry in ESM Appendix 3 did not show any time-trends, which provides some support to our assumption in our Cox proportional hazard models that the dependent variable and the calendar time of generic formulary listing were uncorrelated.

References

1. Fisher LD, Lin DY. Time-dependent covariates in the Cox proportional-hazards regression model. Annu Rev Public Health. 1999;20:145-157. doi:10.1146/annurev.publhealth.20.1.145

2. Hosmer Jr DW, Lemeshow S, May S. Applied Survival Analysis: Regression Modeling of Time-to-Event Data. 2 edition. Hoboken, N.J: Wiley-Interscience; 2008.
