# Supplementary material for: The Impact of Tiered-Pricing Framework on Generic Entry in Canada
Source: Int J Health Policy Manag. 2020 Nov 16;11(6):768–76. doi: 10.34172/ijhpm.2020.215 (PMC9309918; doi:10.34172/ijhpm.2020.215)
Supplement: Supplementary file 3 — contains Figures S2-S5 and Tables S1-S3. [file ijhpm-11-768-s003.pdf]

Supplementary file 3

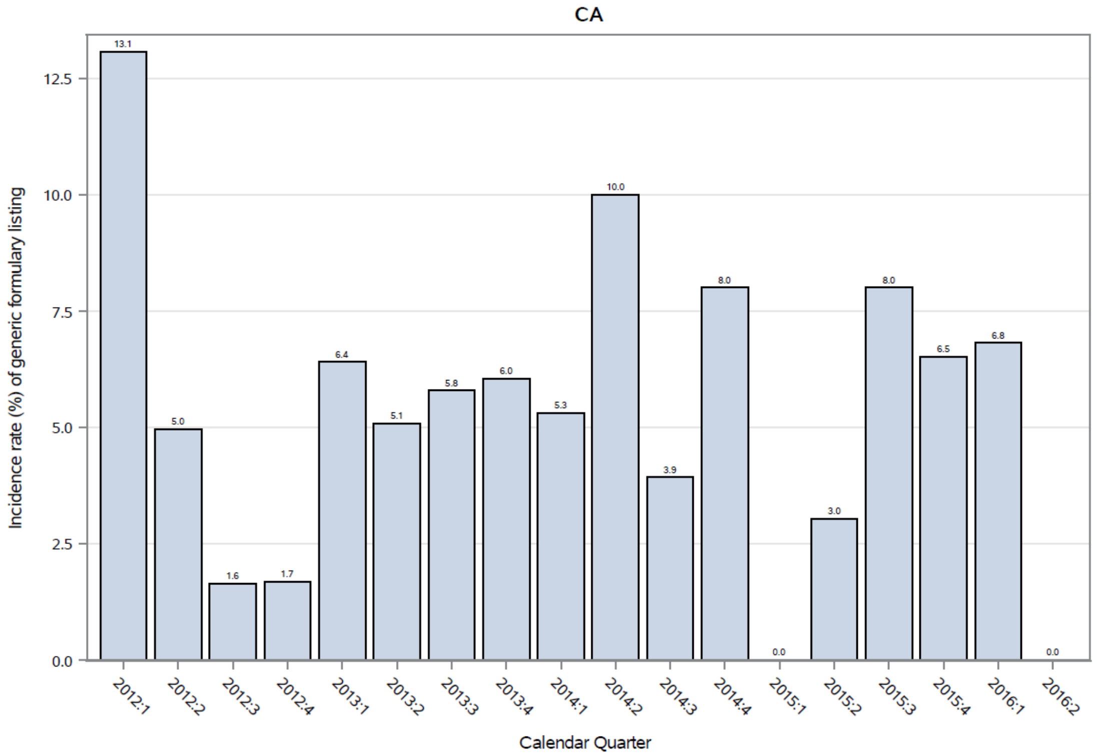

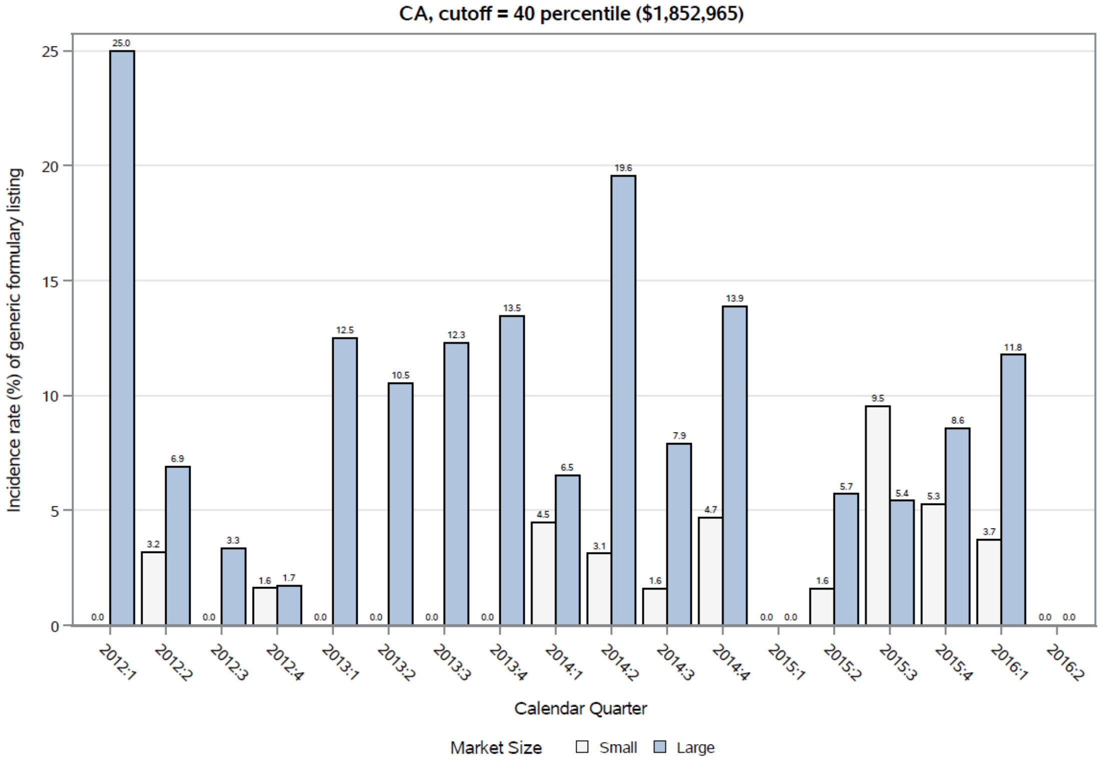

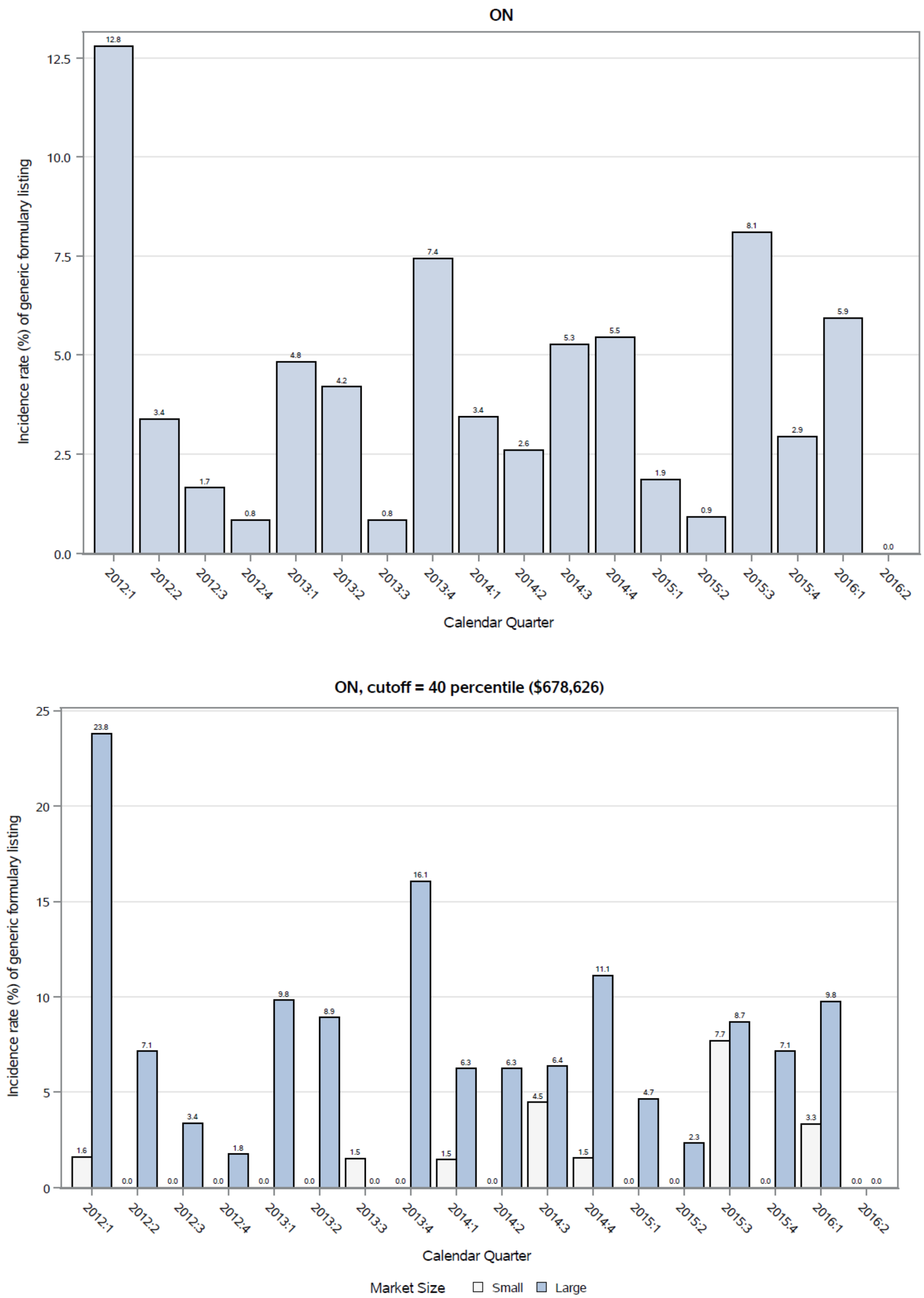

Figure S2. Incidence Rate of First Generic Formulary Listing in Canada and Ontario. Abbreviations: CA, Canada; ON, Ontario.

Table S1. Model Parameters

Percentile for market size	Variable	Level	CA			ON		
			Estimate	SE	P-value	Estimate	SE	P-value
40%	Policy Period	TPF	1.338	0.473	0.005	1.307	0.660	0.048
40%	Market Size	Large	2.009	0.440	0.000	2.605	0.603	0.000
40%	Policy Period× Market Size	TPF x Large market	-1.365	0.532	0.010	-1.560	0.709	0.028
50%	Policy Period	TPF	0.944	0.344	0.006	0.953	0.533	0.074
50%	Market Size	Large market	1.622	0.323	0.000	2.566	0.482	0.000
50%	Policy Period× Market Size	TPF x Large market	-1.101	0.435	0.011	-1.214	0.600	0.043
Abbreviations: CA, Canada; ON, Ontario; SE, standard error; TPF, tiered pricing framework.								

Table S2. Proportional Assumption Testing Using Weighted Schoenfeld Residuals

Percentile for market size	Variable	CA		ON	
		Correlation*	P value	Correlation*	P value
40%	Policy Period	0.140	0.131	0.083	0.443
40%	Market Size	0.062	0.490	0.018	0.862
40%	Policy Period× Market Size	-0.050	0.584	-0.055	0.606
40%	_Global_		0.138		0.696
50%	Policy Period	0.084	0.382	-0.045	0.680
50%	Market Size	-0.090	0.345	-0.130	0.211
50%	Policy Period× Market Size	0.044	0.644	0.066	0.525
50%	_Global_		0.090		0.494
*Correlation between scaled Schoenfeld residuals and time<br>Abbreviations: CA, Canada; ON, Ontario.					

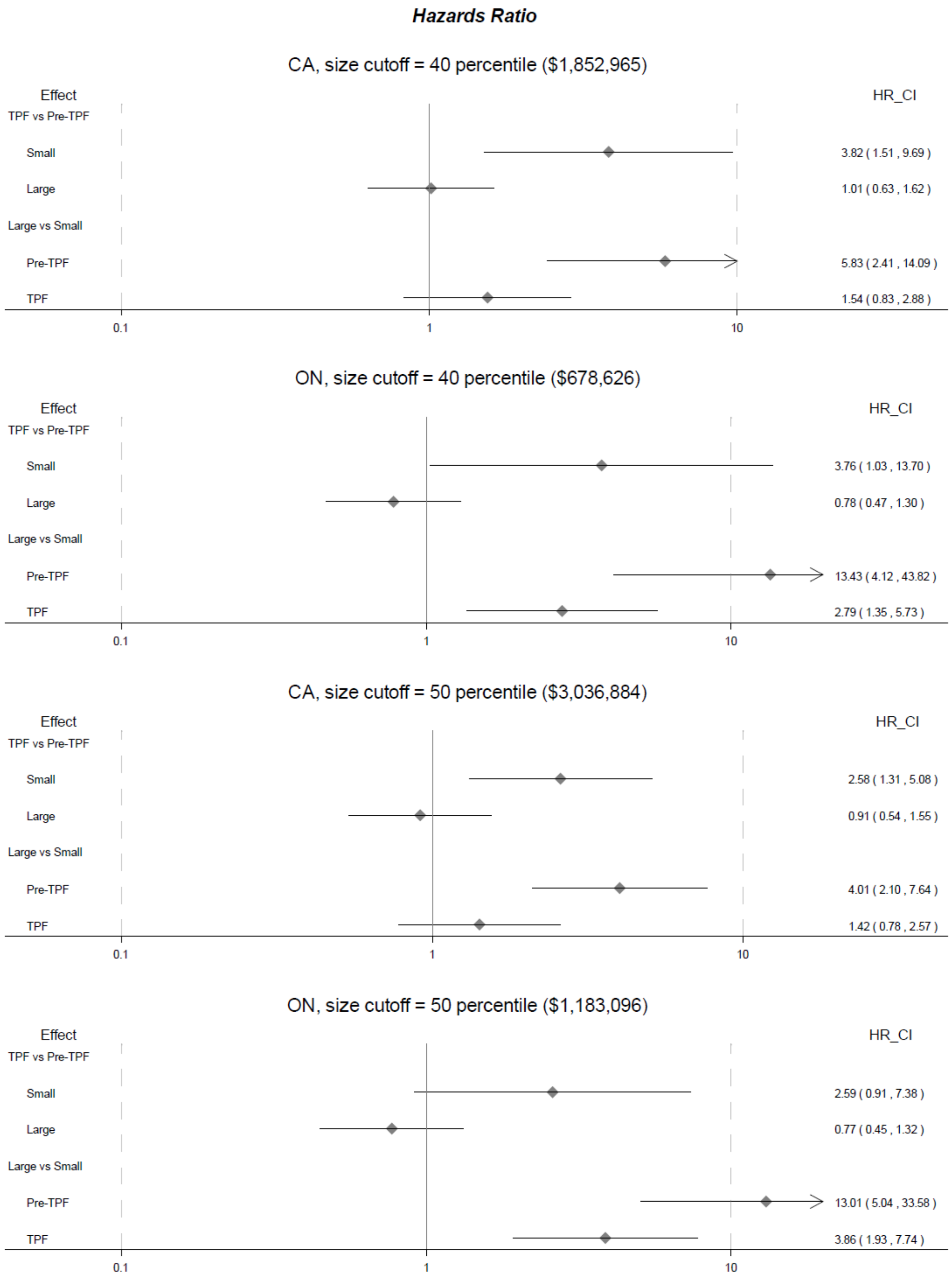

Figure S3. Hazards Ratios From Cox Proportional Hazards Models With Time-Varying Covariates Adjusting for Route/Dosage Formulation. Abbreviations: TPF, tiered pricing framework; CA, Canada; ON, Ontario; HR, hazard ratio; CI, confidence interval.

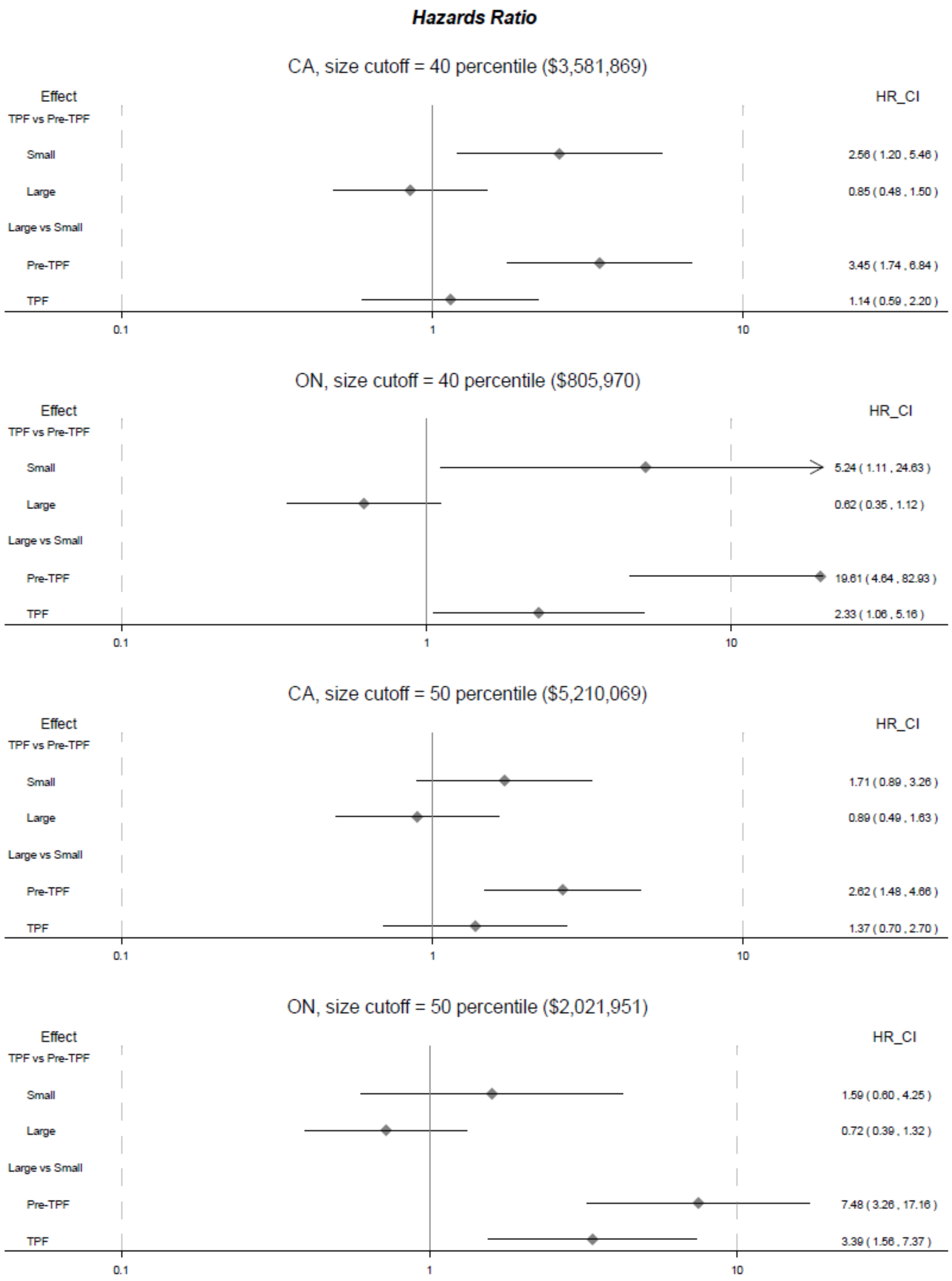

Figure S4. Hazards Ratios From Cox Proportional Hazards Models With Time-Varying Covariates Among the Markets With Oral-Solid Formulation.

N=139 markets in Canada and N=145 markets in Ontario. Abbreviations: TPF, tiered pricing framework; CA, Canada; ON, Ontario; HR, hazard ratio; CI, confidence interval.

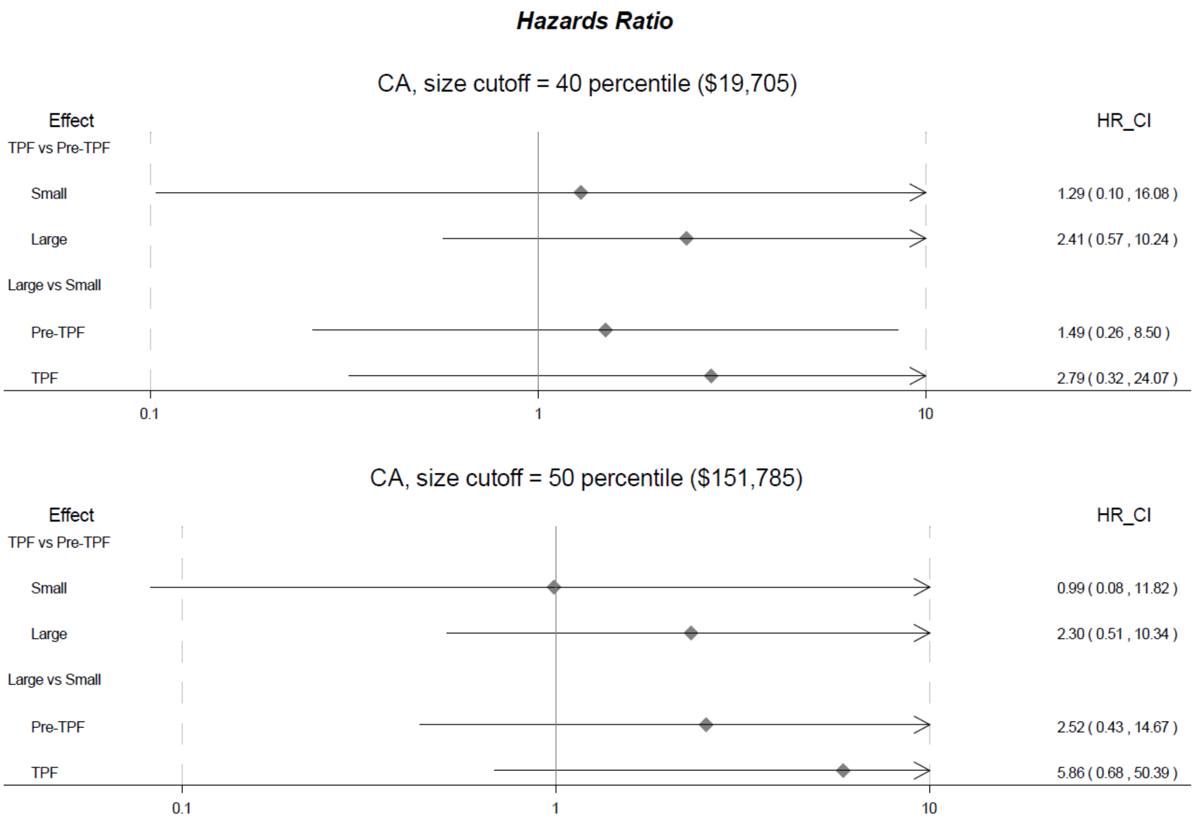

Figure S5. Hazards Ratios From Cox Proportional Hazards Models With Time-Varying Covariates Among the Markets With Non-Oral-Solid Formulation (N = 50).

The analysis was not conducted for Ontario due to the small sample size. Abbreviations: TPF, tiered pricing framework; CA, Canada; HR, hazard ratio; CI, confidence interval.

Table S3. The Second and Third Generic Entry Among Markets With First Generic Entry by Policy Periods In Canada and Ontario.

Province	Period	First generic entry	Second generic entry		Third generic entry	
		N	N (%)	Mean time to entry from first generic entry, months (SD)	N (%)	Mean time to entry from second generic entry, months (SD)
Canada	Pre-TPF	61	41 (67)	3.01 (5.66)	30 (49)	1.26 (2.42)
Canada	TPF	51	33 (65)	1.25 (2.88)	29 (57)	0.75 (1.60)
Ontario	Pre-TPF	48	29 (60)	2.94 (6.90)	22 (46)	0.79 (2.11)
Ontario	TPF	39	29 (74)	0.44 (0.77)	25 (64)	0.32 (0.95)
Abbreviations: TPF, Tiered Pricing Framework; SD, standard deviation.						
